# Allergy-related disorders (ARDs) among Ethiopian primary school-aged children: Prevalence and associated risk factors

**DOI:** 10.1371/journal.pone.0204521

**Published:** 2018-09-25

**Authors:** Nezar Mehanna, Nader Mohamed, Moges Wordofa, Dessie Abera, Abiyot Mesfin, Mistire Wolde, Kassu Desta, Aster Tsegaye, Bineyam Taye

**Affiliations:** 1 Department of Biology, Colgate University, Hamilton, New York, United States of America; 2 Department of Medical Laboratory Sciences, Addis Ababa University, Addis Ababa, Ethiopia; Massachusetts General Hospital, UNITED STATES

## Abstract

**Background:**

There has been a noticeable increase in the prevalence of allergy-related disorders (ARDs) in the modern era. Urbanization is believed to be a major environmental risk factor for the onset of ARDs but data from low- to middle-income countries is limited.

**Objective:**

Our purpose was to assess the prevalence of ARDs and atopy among a population of rural Ethiopian school children and identify environmental and lifestyle factors associated with such disorders.

**Methods:**

We performed a cross-sectional study on 541 school-children. An interviewer-led questionnaire administered to the mothers of each participant provided information on demographic and lifestyle variables. Questions on allergic disease symptoms were based on the International Study of Asthma and Allergies in Children (ISAAC) core allergy and environmental questionnaire. Skin prick test for common allergens German cockroach *(Blattella germanica)* and dust mite (*Dermatophagoides)* was performed to define atopy. Multiple logistic regression analyses were performed to determine the odds ratio between ARDs and atopy with specific environmental and lifestyle habits.

**Results:**

541 children responded to the survey questions: the majority of participants were female (60.3%) and aged 10–15 years-old. The prevalence of any ARD was 27%, while the rates of ever-having eczema, rhinitis, and wheeze was found to be 16.8%, 9.6%, and 8.6% respectively. Only 3.6% (19 school-children) tested positive for any skin sensitization. Analysis of associated factors for ARDs found that a family history of allergic disorders (AOR: 2.80; p-value<0.01), use of insecticides (AOR: 2.05; p-value<0.01), and wearing open-toed shoes (AOR: 2.19; p-value = 0.02) were all significantly associated factors. Insecticide use, river-bathing, and infection with intestinal parasites were found to be significantly associated factors for atopy. Other potential risk factors such as frequent use of soap, bacterial infection, and household crowding had no statistical significance.

**Conclusion:**

Our study suggests that the prevalence of skin sensitization and ARDs in rural populations of developing countries is still relatively low. We identified several possible risk factors for further investigation. Overall, the significance of identified risk factors appears to indicate that genetic predisposition and exposure to environmental pollution are more important to the etiology of ARDs and atopy than specific lifestyle behaviors.

## Introduction

The prevalence of atopy and allergy-related disorders (ARDs), such as eczema, wheeze, asthma, and rhinitis, has risen over the last three decades and continues to be a major public health concern[1]. Current data from the World Allergy Organization (WAO) estimates that sensitization to common allergens is present in 25%-35% of the general population and an estimated 300 million people worldwide are affected by asthma [2]. The burden of ARDs has been shown to disproportionately affect children. Asthma is among the top ten chronic conditions for children aged 5–14 years old [3]. Similarly, atopic dermatitis is prevalent in 15%-20% of children but only 1–3% of adults [4].

Multi-center studies have revealed a striking difference in the prevalence of ARDs between populations in developed and underdeveloped countries [5]. While incidence rates have been shown to be stabilizing in some high-income countries [6, 7], low-to-middle income nations are still facing the brunt of the so-called ‘*allergy epidemic’*—with incidence rates expected to increase in the coming years [5, 8, 9]. However, the majority of published data on allergies has come out of the United States and Western Europe; there is a relative dearth of information about the prevalence of ARDs in populations of Sub-Saharan Africa. Furthermore available data on prevalence rates of ARDs in African countries is largely outdated. ISAAC phase 3 studies have reported that among African nations the prevalence of eczema and asthma is highest in South Africa (20.3% and 13.3%, respectively) and Kenya (21.2% and 15.2%, respectively) [7, 10]. In 2003, Hailu et al. reported the prevalence of rhinitis in Ethiopia to be 15.3% [11], while in 2006 the prevalence of asthma in Ethiopia was estimated to be 9.1% [7].

Allergic diseases are extremely common, yet the etiology of such disorders remains a mystery. A ‘western’, more urban lifestyle is often cited as a major risk factor for the development of allergic disorders [12–15]. This is evidenced by the difference in incidence rates between developed and developing countries as well as findings that prevalence rates vary between urban and rural settings within a single country [14, 16–18]. Environmental factors such as diet, infections, and air pollution have also been identified as playing a possible role in the development of ARDs [4, 19]. Previous studies often contradict each other on the significance of certain risk factors. More data is needed to support the existing hypotheses in current literature. Ethiopia presents an ideal study setting to further elucidate the role of urbanization in relation to allergies as it is a rapidly developing low-income nation and is predicted to reach status as a middle-income country in 2025.

This study aimed to examine the prevalence of allergy-related disorders (categorized as eczema, wheeze, and rhinitis) and atopy (defined as skin sensitization to common allergens) among a population of rural school children in Sululta town, Ethiopia. We report on extensive demographic and lifestyle variables to help identify risk factors associated with ARDs and atopy. To our knowledge, this is the first paper in recent years to report the prevalence of a range of allergic disorders and associated factors within Ethiopia conducted outside the capital of Addis Ababa.

## Methods

### Study setting and design

This cross-sectional study featured 541 school children aged 5–14 years old and was conducted in Sululta town, Ethiopia from April 2017 to August 2017. The town of Sululta is located approximately 35 km from the Ethiopian capital of Addis Ababa. The region has an altitude of 2450m above sea level and average temperatures are in the range of 15°–18° Celsius. The town is estimated to have a population of 49,000. Sululta town is semi-urban; subsistence farming is still practiced but a portion of the population has shifted to daily labor.

### Study subjects

We performed random cluster sampling to select four schools for participation in our study out of a possible nine governmental schools and 28 private schools. The study populations were all school children ages 5–14 years in the selected primary public schools. Our study participants were apparently healthy children attending primary school; they came from comparable socioeconomic backgrounds and resided in the same general environment. It is unlikely the children experienced a large degree of variation in regards to their general exposure to pathogens and access to health care. Our researchers visited each school prior to the beginning of data collection to inform teachers and parents about the goal and nature of the study.

### Questionnaire

After obtaining consent from the child and their legal guardian, a questionnaire was completed through a data collector-administered interview. Each question was translated from English to Amharic, explained in further detail if necessary, and written down on the questionnaire form by the data collectors. The questionnaire was designed based on the standardized rules of the International Study of Asthma and Allergies in Childhood (ISAAC). The first page established demographic characteristics by asking the parent about the student’s age, grade, and sex. Socioeconomic status (SES) was determined by asking about parental occupation, parental education level, monthly income, and tobacco use (*Is there anyone who smokes cigarettes at home*?*)*.

The second part of the questionnaire focused on ascertaining the prevalence of asthma, eczema, and rhinitis among the study participants. Eczema questions included ‘*Has the child experienced an itchy rash in the last 12 months*?’ as well as a follow-up question inquiring about the affected area. For asthma, we asked *“Has the child experienced wheezing in the last 12 months*?*’*, the number of wheezing attacks within the past year, and *“Has a diagnosis of asthma been confirmed by a doctor*?*”* For rhinitis: *“Has the child experienced persistent sneezing attacks and runny nose not related to cold or flu in the last 12 months*?*”* and *“Has the child had hay fever in the last year*?*”* Parental history of eczema, asthma, or rhinitis was also asked by directing the same questions at the mother and father.

The remaining questions established lifestyle habits we hypothesized could be possible risk factors for ARDs or atopy. Among these were variables related to household crowding, fuel use, methods of waste disposal, animals owned, the material of the walls and roof in each child’s home, and the family’s source of drinking water. The English and Amharic version of the questionnaires are supplied as an online supplementary document (S1 Appendix).

### Stool collection and examination

A stool sample was collected from each study participant to analyze the presence of intestinal parasites and Helicobacter pylori (*H*. *Pylori*). To ensure a high level of sensitivity for the detection of intestinal parasites each sample was subjected to three parasitological tests: direct wet mount, Kato Katz technique, and ethyl-ether sedimentation. Direct wet mount of the sample was performed using a 0.85% saline solution. For Kato Katz technique 41.7mg of sieved fecal material was placed on the slide by using a standardized template. Cellophane soaked overnight in methylene blue glycerol solution was placed over the sample before being pressed down and read. In the sedimentation technique, one gram of fecal matter was dissolved in 8mL of formalin and vortexed before being filtered through a gauze mesh. The resulting solution was transferred back to the centrifuge tube and combined with 8mL of ethyl acetate, mixed thoroughly, then centrifuged at 2000rpm for two minutes. Presence of *H*. *Pylori* was determined using rapid stool antigen test kits (Cortez Diagnostics California, USA). Each stool sample was collected at the school site. Direct mount, Kato Katz, and *H*. *pylori* testing were completed at a temporary field lab at the school. Stool samples were preserved with 5mL SAF per gram and transferred back to Addis Ababa University, College of Health Sciences, and Department of Medical Laboratory Sciences where the sedimentation technique was performed.

### Atopic sensitization

Skin prick test (Imunotek Madrid, Spain) was performed using two common allergens: German cockroach *(Blattella germanica)* and dust mite (*Dermatophagoides)*, previously found to be common in an Ethiopian population. Histamine and saline were used as positive and negative controls, respectively. A pipette drop of each solution was placed on the volar surface of the forearm and the dermis was pierced using a metal lancet. After 15 minutes, resulting wheals greater than 3mm in diameter were recorded as positive results.

### Statistical analysis and categorical definitions

All statistical analysis was done with IBM SPSS 24. We categorized a positive response indicating a child experienced wheezing in the last 12 months as the participant suffering from wheeze. ‘Asthma’ was a separate category and only contained children who had been diagnosed with asthma by a medical professional. If the mother or guardian answered in the affirmative to questions of their child having multiple rashes in the last 12 months or being diagnosed with eczema, the participant was categorized as positive for eczema. Rhinitis category included positive responses to the question ‘has the child had persistent sneezing and runny nose that was not cold or flu’ and ‘has the child been diagnosed with hay fever’. Due to sample size constraints, we opted to create an “any ARD” category, which included all participants positive for asthma, wheeze, eczema, or rhinitis. Similarly, the prevalence of sensitization was low so it was necessary to create an “any atopy” category for participants who were positive for either German cockroach or dust mite. Logistic regression was performed to determine the odds ratio for any ARDs or any atopy against possible associated factors. Multivariate analysis was conducted to account for possible confounding variables. Statistical significance was defined as a p-value<0.05 and a 95% confidence interval for each odds ratio was calculated.

### Ethical approval

The study was approved by the Departmental Research and Ethics Review Committee (DRERC) of Addis Ababa University, Department of Medical Laboratory Sciences, Ethiopia. Written, informed consent was obtained from the mothers or guardians after they have been clearly briefed about the study. Children were also requested to give assent and were informed of their right to refuse to participate in the study and to withdraw at any time during the study without jeopardizing their right to access other health services. invasive procedures such as skin prick testing was fully explained to parents and children and was carried out using sterile disposable materials.

## Results

Of the 541 school-children surveyed, 96.8% participated in a skin prick test and 98.7% provided a stool sample. The study population had a mean age of 10.81 ± 2.59 and 60.3% (324) of the students were female. The majority of participants resided in rural areas near their school (76%), had a family size greater than 5 people (59.7%), and lived off a monthly income of 1000–2000 Ethiopian birr (52.2%). Over three-fourth of the study participants had been dewormed in the last 6 months (80.9%) but 30.7% of children still tested positive for presence of intestinal parasites. (Table 1).

**Table 1:** Selected demographic and lifestyle characteristics of school children and their guardians in Sululta town Ethiopia (*N = 541*).

Variables	Frequency (%)
**Sex**	
Female	324 (60.3)
Male	213 (39.7)
**Place of Residence**	
Rural	406 (76)
Urban	128 (24)
**Age**	
4–9	162 (30.1)
10–15	377 (69.9)
**Family Size**	
1–4	209 (38.6)
>5	323 (59.7)
**Maternal Occupation**	
Housewife	311 (57.5)
Farmer	10 (1.8)
Office	68 (12.6)
Other	152 (28.1)
**Maternal Education**	
Illiterate	280 (51.8)
Read and Write	89 (16.5)
Formal Education	172 (31.8)
**Paternal Occupation**	
Office	160 (29.6)
Farmer	50 (9.2)
Merchant	182 (33.6)
Other	121 (22.4)
Unemployed	28 (5.2)
**Paternal Education**	
Illiterate	92 (17)
Read and Write	164 (30.3)
Formal Education	285 (52.7)
**Deworming**^a^	
Dewormed	435 (80.9)
Non-Dewormed	103 (19.1)
**Any parasites** ^b^	
Yes	299 (30.7)
No	676 (69.3)
**Income Groups**^c^	
>1000	57 (15.9)
1000–2000	187 (52.2)
>2000	114 (31.8)
**Tobacco Use** ^d^	
Smokes	43 (8.3)
No Smoking	476 (91.7)

^a^ Deworming status was defined as treatment with 600mg albendazole within the last 6 months

^b^. Any parasites defined if any parasite infection was detected during stool examinations

^c^ Income is given as Ethiopian birr per month; 1 Ethiopian birr = 0.036 US dollar

^d^ Smoking question refers to household members and not the child

### Prevalence of allergic disorders and skin sensitization

Among the 541 study participants, the presence of ARDs was observed in 146 cases, 27% of the study population. Eczema had the highest prevalence of the allergic disorders (16.8%, 87 cases); rhinitis was the second highest reported with 9.5% of children reporting symptoms. Clinician diagnosed asthma was reported in only 17 study participants, but self-reported wheeze was more prevalent (8.6%, 46 cases). For atopy, the overall prevalence of any skin sensitization was 3.6% (19 cases), with sensitization to German cockroach (2.9%) being greater than that of dust mite (1.7%) (Table 2).

**Table 2:** Prevalence of self-reported allergic disorders and atopy among school children in Sululta, Ethiopia.

Variables	Frequency (%)	95% CI^C^
**Any Allergic Disorders** ^a^		
Yes	146 (27)	23–31
No	395 (73)	
**Asthma**		
Yes	17 (3.2)	2–5
No	510 (96.8)	
**Eczema**		
Yes	87 (16.8)	14–20
No	431 (83.2)	
**Rhinitis**		
Yes	50 (9.5)	7–13
No	474 (90.5)	
**Wheeze**		
Yes	46 (8.6)	6–11
No	487 (91.4)	
**Any Atopy**^b^		
No Reactivity	505 (96.4)	
Allergic	19 (3.6)	2–6
**Dust Mite**		
No reactivity	515 (98.3)	
Allergic	9 (1.7)	1–3
**German Cockroach**		
No Reactivity	508 (97.1)	
Allergic	15 (2.9)	1.6–4.7

^a^ Any allergic disorder was defined as any positive response for symptoms of either asthma, eczema, hay fever, or wheezing using ISAAC questionnaire

^b^ Presence of atopy indicates a reaction to either the skin prick test for German cockroach or dust mite producing a wheal greater than 3mm in diameter

^c.^ 95% Confidence interval (CI)

### Risk factors for all allergy-related disorders

Table 3 shows the crude and adjusted odds ratio for a list of potential risk factors and their association with ARDs. The prevalence of allergic disorders was significantly lower in the 4–9 age group than 10-15-year-olds (21.6% vs 29.2%, p-value = 0.04), but there was no significant difference in the prevalence of allergic disorders between gender, place of residence, or income levels. However, ARDs were significantly associated with a family history of allergic disorders (AOR: 2.80, p-value<0.01) and the presence of atopy (AOR: 3.54, p-value = 0.01). The largest adjusted odds ratio was observed for ‘type of toilet’ category, with use of a modern flush toilet over a traditional pit or field latrine being a significant risk factor (AOR: 5.95, p-value = .01). Wearing sandals as opposed to closed-toe shoes (AOR: 2.19, p-value = 0.02) and the use of insecticides (AOR: 2.05, p-value <0.01) were also significantly associated with a higher prevalence of ARDs. Income level, household crowding, *H*. *pylori* infection, and parasitic infection were found to be non-significant factors. In our univariate analysis deworming was found to be a significant protective factor for allergic disorders (COR: 0.53, p-value<0.01), but after adjustment for confounders the p-value increased to 0.22.

**Table 3:** Crude and adjusted odds ratio for the association of select risk factors to ARDs^a^ among school-aged children in Sululta town, Ethiopia.

Variable	Allergy (%)	No allergy (%)	COR (95% CI)	p-value	AOR^b^ (95% CI)^c^	p-value
**Age**						
4–9	35 (21.6)	127 (78.4)	0.67 (0.43–1.03)	0.07	0.55 (0.31–0.97)	0.04
10–15	110 (29.2)	267 (70.8)	1		1	
**Gender**						
Female	83 (25.6)	241 (74.4)	1			
Male	61 (28.6)	152 (71.4)	1.165 (0.80–1.71)	0.44	0.76 (0.46–1.26)	0.28
**Residence**					1	
Rural	111 (27.3)	295 (72.7)	1			
Urban	34 (26.6)	94 (73.4)	0.96 (0.61–1.51)	0.86	1.22 (0.66–2.26)	0.53
**Income**^d^					1	
<1000	15 (26.3)	42 (73.7)	0.69 (0.34–1.4)	0.30	0.64 (0.30–1.37)	0.25
1000–2000	52 (27.8)	135 (72.2)	0.74 (0.38–1.17)	0.24	0.666 (0.38–1.17)	0.16
>2000	39 (34.2)	75 (65.8)	1		1	
**Family History**^e^						
Yes	37 (43.0)	49 (57.0)	2.40 (1.49–3.87)	<0.01	2.80 (1.63–4.80)	<0.01
No	109 (24.0)	346 (76.0)	1		1	
**Crowding** ^f^						
0.4–1.8	56 (28.9)	138 (71.1)	1		1	
1.9–2.8	35 (21.9)	125 (78.1)	0.70 (0.42–1.12)	0.14	0.65 (0.39–1.08)	0.09
>2.8	44 (27)	119 (73)	0.91 (0.57–1.45)	0.70	0.96 (0.59–1.59)	0.88
**Atopy**						
Positive	9 (47.4)	10 (52.6)	2.42 (0.96–6.08)	0.06	3.54 (1.28–9.76)	0.01
Negative	137 (27.1)	368 (72.9)	1		1	
**Insecticides**						
No Use	82 (24)	259 (76)	1		1	
Use	64 (32)	136 (68)	1.49 (1.10–2.19)	0.04	2.05 (1.20–3.49)	<0.01
**H. Pylori**						
No	125 (27)	338 (73)	1		1	
Infection	13 (22.4)	45 (77.6)	1.28 (0.67–2.45)	0.46	1.17 (0.54–2.54)	0.69
**Animals**						
Animals	93 (25.3)	275 (74.7)	1		1	
No animals	53 (30.6)	120 (69.4)	1.13 (0.75–1.69)	0.57	1.24 (0.71–2.17)	0.44
**Deworming** ^g^						
Dewormed	106 (24.4)	329 (75.6)	0.53 (0.34–0.83)	<0.01	0.70 (0.39–1.24)	0.22
None	39 (37.9)	64 (62.1)	1		1	
**Parasites**^h^						
No Infection	105 (27)	284 (73)	1		1	
Infection	41 (30.1)	95 (69.9)	1.17 (0.76–1.79)	0.48	1.41 (0.82–2.40)	0.21
**Toilet Type**						
Toilet	9 (40.9)	13 (59.1)	2.51 (0.91–6.92)	0.07	5.95 (1.51–23.47)	0.01
Pit	119 (26.9)	324 (73.1)	1.33 (0.74–2.41)	0.34	1.54 (0.69–3.65)	0.29
Field	16 (21.6)	58 (78.4)	1		1	
**Soap Use**						
Always	41 (31.3)	90 (68.7)	1.33 (0.86–2.05)	0.19	1.14 (0.67–1.93)	0.63
Sometimes	99 (25.5)	289 (74.5)	1.10 (0.42–2.88)	0.85	0.68 (0.23–2.02)	0.49
No Soap Use	6 (27.3)	16 (72.7)	1		1	
**Fruit Washing**						
Always	34 (29.6)	81 (70.4)	0.54 (0.26–1.12)	0.097	0.65 (0.30–1.41)	0.25
Sometimes	89 (24.5)	275 (75.5)	0.41 (0.21–0.80)	0.01	0.44 (0.22–0.90)	0.03
Never	18 (43.9)	23 (56.1)	1		1	
**Shoe Habits**						
Closed toe	36 (19.8)	146 (80.2)	1		1	
Sandals	58 (29)	142 (71)	1.66 (1.03–2.66)	0.04	2.19 (1.11–4.29)	0.02
**Smoking**						
Tobacco Use	11 (25.6)	32 (74.4)	1		1	
No Tobacco Use	128(26.9)	348 (73.1)	1.07 (524–2.186)	0.853	0.734 (.294–1.881)	0.532

^a^ ARDs were defined as positive response to symptoms of asthma, rash, hay fever, or wheeze

^b^ Variables were adjusted for place of residence, sex, age, maternal occupation, income, and other possible cofounders

^c.^ 95% Confidence interval

^d.^ Income is given as Ethiopian birr per month; 1 Ethiopian birr = 0.036 US dollars”

^e.^ Family history of allergy was defined positive response to symptoms of asthma, rash, hay fever, or wheeze by mothers or fathers of the child

^f.^ Crowding was calculated as family size divided by amount of bedrooms in household

^g.^ Dewormed indicated treatment with 600mg albendazole within the last 6 months

^h.^ Identification of a parasite from any of three laboratory tests was coded as positive for intestinal parasites

### Risk factors for atopy

Crude and adjusted odds ratios for possible factors associated with atopy are shown in Table 4. Use of insecticides and a family history of ARDs were both significant risk factors (AOR: 7.47, p-value = 0.01 and AOR: 3.24, p-value = 0.02, respectively). Prevalence of atopy was also significantly higher among those infected with intestinal parasites (AOR: 3.52, p-value = 0.03). Children who yes to the question of ‘do you bathe in the local river?’ had the highest adjusted odds ratio among the statistically significant factors (AOR: 8.72, 95% CI: 2.04–37.28, p-value<0.01). Nail trimming was a protective habit with untrimmed nails carrying 4.54 greater odds of atopy (95% CI: 1.50–13.80; p-value < .01). Soap use, income level, place of residence, and deworming status were found to be not significant (Table 3).

**Table 4:** Crude and adjusted odds ratio for the association of select lifestyle variables and atopy^a^ among the study population of children in Sululta, Ethiopia.

Variable	Reactive (%)	No reaction (%)	COR (95% CI)	p-value	AOR^b^ (95% CI)	p-value
**Age**						
4–9	7 (4.5)	148 (95.5)	1.39 (0.54–3.62)	0.49	1.42 (0.50–4.02)	0.51
10–15	12 (3.3)	355 (96.7)	1			
**Gender**						
Female	7 (2.3)	303 (97.7)	1			
Male	12 (5.7)	198 (94.3)	2.62 (1.02–6.78)	0.04	3.02 (1.08–8.48)	0.03
**Residence**						
Rural	14 (3.6)	379 (96.4)	1.11 (0.36–3.43)	0.86	1.03 (0.30–3.51)	0.96
Urban	4 (3.2)	120 (96.8)	1			
**Family History**^c^						
Yes	7 (8.2)	78 (91.8)	3.19 (1.22–8.36)	0.02	3.24 (1.19–8.79)	0.02
No	12 (2.7)	427 (97.3)	1			
**Income**^d^						
<1000	1 (1.8)	55 (98.2)	0.49 (0.05–4.45)	0.53	0.33 (0.02–5.08)	0.42
1000–2000	9 (4.9)	175 (95.1)	1.39 (0.42–4.62)	0.59	2.39 (0.58–9.58)	0.22
>2000	4 (3.6)	108 (96.4)	1		1	
**Crowding**^e^						
0.4–1.8	8 (4.2)	181 (95.8)	1.69 (.50–5.72)	0.40	4.916 (1.08–22.48)	0.04
1.9–2.8	7 (4.5)	148 (95.5)	1.81 (.52–6.31)	0.35	0.517 (0.07–3.62)	0.51
>2.8	4 (2.5)	153 (97.5)	1			
**Insecticide Use**						
No use	7 (2.1)	323 (97.9)	1		1	
Used	12 (6.2)	182 (93.8)	3.04 (1.8–7.86)	0.02	7.47 (1.55–35.97)	0.01
**H. Pylori**						
Not present	18 (4)	431 (96)	1		1	
H. Pylori	1 (1.8)	54 (98.2)	2.26 (.29–17.23)	0.43	0.293 (0.03–2.82)	0.29
**Animals**						
No animals	8 (4.7)	163 (95.3)	1.53 (0.60–3.87)	0.37	3.51 (1.06–11.58)	0.04
Animals	11 (3.1)	342 (96.9)	1		1	
**Deworming**^f^						
Dewormed	15 (3.6)	406 (96.4)	1		1	
Non	4 (4)	96 (96)	1.13(0.37–3.47)	0.83	1.11 (.29–4.22)	0.88
**Parasites** ^g^						
No infection	10 (2.6)	376 (97.4)	1		1	
Parasite	9 (6.7)	125 (93.3)	2.71 (1.08–6.81)	0.03	3.52 (1.15–10.71)	0.03
**Toilet**						
Toilet	1 (4.5)	21 (95.5)	0.79 (0.083–7.42)	0.83	0.78 (0.05–12.58)	0.86
Pit	14 (3.3)	416 (96.7)	0.56 (0.18–1.74)	0.31	0.32 (0.08–1.34)	0.12
field	4 (5.7)	66 (94.3)	1		1	
**Soap Use**						
High Soap Use	6 (4.7)	121 (95.3)	1		1	
Moderate	13 (3.5)	363 (94.2)	0.72 (.27–1.94)	0.52	0.90 (0.30–2.88)	0.90
No Soap Use	0 (0)	21 (100)	0	0.99	0	0.99
**Nail Trimming**						
Trimmed	8 (2.3)	338 (97.7)	1		1	
Not Trimmed	11 (7.1)	143 (92.9)	3.25 (1.38–8.25)	0.01	4.54 (1.50–13.80)	<0.01
**River Bathing**						
No	13 (2.8)	452 (97.2)	1		1	
Yes	6 (10.2)	53 (89.8)	3.94 (1.44–10.79)	<0.01	8.72 (2.04–37.28)	<0.01
**Smoking**						
Tobacco Use	2 (5)	38 (95)	1.38 (0.31–6.22)	0.67	1.42 (0.26–7.91)	0.69
No Use	17 (3.7)	447 (96.30)	1		1	

^a^ Atopy was defined as positive reactions to skin prick test for either German cockroach or dust mite with wheal of minimum 3mm

^b^ Variables were adjusted for place of residence, age group, sex, income level, and deworming status

^c^ Family history of allergy was defined as a positive response to symptoms of asthma, rash, hay fever, or wheeze by mothers or fathers of the child

^d^ Income is given as Ethiopian birr per month; 1 Ethiopian birr = 0.036 US dollars”

^e^ Crowding was calculated as family size divided by amount of bedrooms in household as reported by guardians in the questionnaire

^f^ Dewormed indicated treatment with 600mg albendazole within the last 6 months

^g^ Identification of a parasite from any of three laboratory tests was coded as positive for intestinal parasites

## Discussion

This study expounds upon the modest body of literature investigating the prevalence and associated factors of allergic diseases in Sub-Saharan Africa. In this cross-sectional study, we found the prevalence of skin sensitization and allergy-related disorders in Sululta, Ethiopia to be relatively low. Eczema and rhinitis were found to be the most common ARDs, with atopy having a lower frequency than any ARD. The factors most strongly associated with a higher prevalence of atopy and ARDs were the use of insecticides and a family history of allergic diseases.

We report the prevalence of skin sensitization in Sululta town to be 3.6%. This result is comparable to other studies conducted in rural, underdeveloped areas and is lower than the values seen in more developed, industrialized nations. A New Zealand cohort by Purvis et al. reported the prevalence of skin sensitization to be at 15.8% [20], while studies in rural African communities—such as a previous report from Ethiopia in 2005—put the prevalence of atopy at 4.4%[21]. Our calculated prevalence of ever having ARDs was also similar to other epidemiological studies. A 2007 ISAAC phase III study focusing on populations of 13- to 14-year old children in African nations reported the prevalence of wheeze and asthma in Addis Abba, Ethiopia to be at 9.1% and 2.1%, respectively [22]. Our results show the prevalence of wheeze to be 8.6% and the prevalence of asthma is 3.2%. The same ISAAC study reported a prevalence of eczema at 19% and allergic rhinitis at 9.9%. This matches our values of eczema at 16.8% and rhinitis at 9.5%. Compared to other nations, the prevalence of ARDs in Ethiopia is on the lower end of the spectrum—significantly less than the prevalence of wheeze in the UK which is at 36.8%—but higher than other low-income countries, such as Indonesia, where wheeze prevalence is estimated at 1.6% [5].

Although the causes of allergic diseases are poorly understood, genetic predisposition is thought to be a major factor. In a study based in Korea, the most important risk factor identified for atopic dermatitis was a parental history of allergic disease [23]. In our study, we chose to combine paternal and maternal history of allergies into one parental category. This decision is supported by a previous study that demonstrated paternal and maternal allergic diseases were equally strong determinants of ARDs in children [24]. Based on our results, a family history of allergies is a significant risk factor for all ARDs and atopy, suggesting genetic markers may leave children more susceptible to environmental allergens.

Male sex has also been previously identified as a risk factor for allergies. It has been proposed that the difference in prevalence is caused by hormonal variation that corrects at puberty. Before the onset puberty, males are more at a risk for allergic diseases, but the trend reverses after puberty [24]. Since our study focused on school-children all under the age 15, the higher prevalence of ARDs among males is congruent with this hypothesis. Another interesting finding related to demographic variables was the lower prevalence of ARDs among the 4–9 age group (AOR: 0.55, p-value = 0.04) than the 10- to15-year old participants. This result could be explained by the theory of the ‘atopic march’, where atopic sensitization is more common during early ages and leads to an increase in the incidence of ARDs with age [25] In line with the atopic march, our data did also reveal a higher magnitude of sensitization in the 4–9 than10-15 age group but the difference was not significant. Relatedly, we found that atopy was associated with higher prevalence of ARDs (AOR: 3.54, p-value = 0.01). A comorbidity of allergic diseases is common and has been reported on previously, but this result also fits nicely into the framework of the ‘atopic march’, where early sensitization to common allergens is believed to be a risk factor for later development of allergic disorders [25, 26].

The most popular theory that draws together reported risk factors of allergies into a cohesive mechanism is the hygiene hypothesis. The hygiene hypothesis proposes that infections are protective against allergies [27]. The biological mechanism of the hypothesis is based on the division of the immune system into a Th1 (virus and bacteria) and a Th2 (allergies) branch. The separate branches are counter-regulatory, so we would expect a lack of Th1 stimulation to lead to a proliferation of Th2 cells, thus increasing the individual’s sensitivity to allergies. Applying this logic, we would expect non-dewormed, no soap use, and high crowding to be protective factors for allergic disorders. However, none of these factors were significantly associated with a lower prevalence of either atopy or allergic disorders. Similarly, studies have previously reported *H*. *pylori* infection as a protective factor for atopy [28, 29], but we failed to reproduce this result. In addition, factors that should be protective according to the hygiene hypothesis, such as river bathing or infection with intestinal parasites, were found to be risk factors.

The potential protective effect of intestinal parasites against allergies has been a large part of the investigation into the hygiene hypothesis and has led to the creation of related theories such as the ‘Old friends’ hypothesis that focuses on lack of exposure to parasitic worms and bacteria as the chief cause of increased rates of allergies. However, recent studies still report inconsistent results on whether intestinal infections are a protective factor [13] or a risk factor [30]. We found that infection of intestinal parasites was non-significantly associated with ARDs but significantly associated with a higher prevalence of atopy. The discrepancy in results could be due to the specific species of parasites the host is infected with. In our study population, there were a surprisingly high percentage of children infected with intestinal parasites despite the high rate of deworming. Most children were infected with *Giardia lamblia* and *E*. *Histolytica* while the more common helminths (*Ascaris* and hookworm) made up a lower portion of the parasite burden (not shown). It is possible that deworming treatment is more effective against helminths than protozoa, explaining the unusual distribution of parasites. Such conclusion requires a more in-depth analysis and fall outside the scope of our paper. For now, our finding of a higher prevalence of atopy among infected children supports the idea that intestinal parasites could cause a proliferation of Th2 cells and an increased sensitivity to allergens. Overall, our results reveal no consistent pattern to support the hygiene hypothesis. This may be because all the study participants were still exposed to a moderate level of infectious agents living in Ethiopia despite lifestyle habits considered ‘hygienic’.

A theory more in line with our findings would be the role of environmental pollutants in causing allergic diseases. In a seminal study, Gehring et al. found that exposure to traffic-related air pollution may cause asthma in children [31]. Exposure to air and water pollution could partly explain why river bathing and open-toed shoes were strong indicators of atopy and ARDs in the current study. Previous studies focusing on cohorts of farmers found that pesticides were significantly associated with wheeze and allergic rhinitis [32, 33]. We also found that insecticide use was a significant risk factor for ARDs as well as for atopy (AOR: 7.47, p-value = 0.01). Other studies on farmers reported on the protective effect of livestock exposure to partly explain the rural-urban gradient [34]. Our results, on the other hand, showed that the presence of animals in the household was not significantly associated with ARDs or atopy. This is probably a result of the small sample size of participants in our study reporting the presence of animals in the household. We also failed to show a statistically significant difference in the prevalence of ARDs between participants in urban versus rural areas, which can be contrasted with previous reports showing that urbanization of an area is associated with increased prevalence of respiratory allergic disease [35, 36]. One large scale study of almost 10,000 participants conducted in Mongolia found an increasing rate of sensitization between different levels of urbanization—from 13.6% in villages, 25.3% in rural towns, to 31.0% in the city—giving strong evidence that economic development is directly tied rates of allergic disorders [37]. Our data shows a higher, yet non-significant, prevalence of self-reported allergic disorders (ARDs) among rural inhabitants compared to their urban peers. This could be due to variation in living condition between rural and urban residence. This has been backed from previous epidemiological studies suggesting that in individuals with poor housing conditions (involving increased exposure to dampness, mold, and generally poorer indoor air quality), [38–40] are associated with an increased risk of atopic diseases. We have no data on detailed housing condition, however, a previous study in Ethiopia documented poor housing condition among rural than urban residence [41].

Our findings must still be interpreted with caution. This epidemiological study had to rely on self-reported symptoms of ARDs as a professional diagnosis was not readily available given the nature of health care in the country and hence susceptible to reporting or information bias. Our survey however was based on the widely validated ISAAC symptoms questionnaire [42]. which has been successfully used in Ethiopia among under-five children [21], and in older age groups, [43, 44] which increased the validity of our findings. Moreover, wheezing symptoms in children in the study area are described using a well-known, onomatopoeic Amharic term called “sit-sit” which also increased the validity of our findings. A previous study in Ethiopia on the incidence and prevalence of self-reported allergic outcomes appeared to be positively related to allergen sensitization, suggesting that these are markers of allergy [29]. The panel of allergens used in the skin prick test was limited to two domestic allergens previously found to be common in an Ethiopia population [43], which may not cover the sensitized allergens profile of any given individual. It is possible our reported prevalence of atopy underestimated the true parameter, yet out prevalence rate of 3.6% is still comparable to other rates of sensitization in rural African communities [21]. Although we did not specifically evaluate maternal ability to differentiate hayfever and asthma symptoms, the question designed to assess hayfever was very specific: *‘Has your child ever (or in the past 12 months) had problems with sneezing or running nose (when not affected by cold or flu)*, *or problems with itchy watery eyes*?

The strengths of this study are that atopy outcome was measured objectively by a skin prick test using domestic allergens previously found to be common in Ethiopia [43]. We also included study subjects randomly with participation rates at each school >90%, thereby minimizing selection bias. The use of a validated ISAAC symptoms questionnaire to measure the outcome and the comprehensiveness of the risk factors evaluated in this study was an additional strength.

In conclusion, we report that the prevalence of skin sensitization in Ethiopia is low—and while the prevalence of allergic disorders is higher than other low-income countries—it is still relatively small compared to developed nations. ARDs and atopy were strongly associated with a family history of allergies and insecticide use. For atopy, river bathing was a risk factor which in part suggesting evidence for the role of pollution in the etiology of allergic diseases. While we identified many factors significantly associated with a higher prevalence of atopy and ARDs, more reports are needed to investigate whether these factors are causal. While urbanization and hygienic habits have been well established as an environmental risk for the development of allergy-related diseases, we did not isolate any specific behaviors that support the hygiene hypothesis. This suggests that it is, in fact, a distinct interplay of many different lifestyle changes that contribute to the development of allergies disorders.

## Supporting information

S1 Appendix Questionnaire English and Amharic versions. (PDF)
