# Supplementary material for: Allergy-related disorders (ARDs) among Ethiopian primary school-aged children: Prevalence and associated risk factors
Source: PLoS One. 2018 Sep 25;13(9):e0204521. doi: 10.1371/journal.pone.0204521 (PMC6155548; doi:10.1371/journal.pone.0204521)
Supplement: S1 Appendix — (PDF) [file pone.0204521.s001.pdf]

Annex- VIII: Questionnaire English version

We thank gratefully for your agreement to participate in this study. Now we are going to undertake interview with you and the interview is about general socio demographic characteristics and other health related questions. The information you give us is very essential for this study. Therefore, we politely ask you to give us the right response.

Section 1.SCHOOL, CHILD, FAMILY AND INTERVIEWER IDENTIFICATION

SCHOOL NAME(INCLUDE PHONE NUMBER) ________________	NAME OF CHILD: ________________	NAME OF INTERVIEWER ________________
GRADE OF A CHILD ________________	UNIQUE ID NUMBER [ ]	DATE OF INTERVIEW (dd/mm/aaaa) [/ /]
**Address : Kebele ____**<br>**a)Rural**<br>**b)Urban**	PHONE(FAMILY)	

**1**	What is the sex of the child?	a) Girl (1)	b) Boy (0)	
**2**	When is the child's birthday? (dd/mm/aaaa)	/ / ______	Age ______	*Don't know*
**3**	Weight of the child?	______	Kg	*(Not measured)*
**4**	Height of the child?	______	Meters	*(Not measured)*

1.3 Maternal occupation 1) Civil servants 3) Private organization 5) Daily laborer

2) House wife 4) Farmer 6) Merchant

1.4 Maternal Educational status

0) Illiterate 1) read and write only 2) primary 3) high school 5) higher education

1.5 Paternal occupation 1) Civil servant 3) Merchant 5) Farmer

2) Private organization 4) Daily laborer 6) No work

1.6. Paternal Educational Status

0) Illiterate 1) read and write 2) primary 3) high school 5) higher education

1.7 Family size_________

1.8. Family income/month ___________

Section 3.Allergy Characteristics of a child

G01	Has your child ever had wheezing or whistling in their chest? if you have answered "no" please skip to question 6	Yes	1		WHZL6A
		No	2		
G02	In the last 2 years, has your child had wheezing or whistling in their chest?	Yes	1		WHZT6A
		No	2		
G03	In the last 1 year, has your child had wheezing or whistling in their chest?	Yes	1	→ G04	WHZ6A
		No	2	→ G05	
G04	How many times in the last year has your child had an attack of wheezing?	0	1		WHZFRQ6A
			2		
			3		
			4		
G05	Has your child ever had Asthma?	Yes	1		ASTL6A
		No	2		
G06	In the last 2 years, has your child had Asthma?	Yes	1		ASTT6A
		No	2		
G07	Has your child had Asthma in the last year?	Yes	1	→ G08	AST6A
		No	2	→ G09	

G08	Has this been confirmed by a doctor?	Yes	1		ASTHDR6A
		No	2		
G09	Has your child ever had an itchy skin rash which has affected the skin creases (eg, the folds of the elbow or behind the knees)?	Yes	1		RASHL6A
		No	2		
G10	In the last 2 years, has your child had an itchy skin condition affecting the skin creases (front of the elbow, behind the knees, the front of the ankles, around the neck, or around the eyes?	Yes	1		RASHT6A
		No	2		
G11	In the last 1 year, has your child had an itchy skin condition affecting the skin creases (front of the elbow, behind the knees, the front of the ankles, around the neck, or around the eyes?	Yes	1	→G11A	RASH6A
		No	2	→ G12	

G11A	If yes, has this rash affected any of the following places? **(Multiple Answers possible)**	The elbow folds	1	RASHL6AA
			2	
		Behind the knees	1	RASHL6AB
			2	
		In front of the ankles	1	RASHL6AC
			2	
		Under the buttocks	1	RASHL6AD
			2	
		Around the neck	1	RASHL6AE
			2	

		Around the eyes/ears	1	RASHL6AF
			2	

G12	Has your child ever had hay fever or persistent sneezing attacks?	Yes	1		HAYFL6A
		No	2		
G13	In the last 2 years, has your child had hay fever or persistent sneezing with sneezing or running nose (excluding colds or flu), or problems with itchy watery eyes?	Yes	1		HAYFT6A
		No	2		
G14	In the last year, has your child had hay fever or persistent sneezing with sneezing or running nose (excluding colds or flu), or problems with itchy watery eyes?	Yes	1		HAYF6A
		No	2		

		No			
G15	For identifying the deworming status;<br>Has your child taken any de-worming medication in the last 6 months?	Yes	1		DEWOR6A
		No			

G16	Is there anyone who smokes cigarettes in your home?	Yes	1	→ G29A	HCIGR6A
		No	2	→ G30	
G16A	If yes, please write the total number of people who smoke cigarettes in the home where the child living?	[ ]			HCIGRN6A
G17	What does your child sleep on?	Bed	1		CHSLP6A
		Medeb	2		
		Floor	3		
		‘Jibba’	4		

		'Sigaja'	5	
		Other (Specify)	9	
G17	What is your child's bed made of?	Iron metal	1	CHBED6A
		Wood	2	
		Flat metal	3	
		Rope	4	
		leather	5	
		No bed	6	
		Other (Specify)	9	
G18	What is your child's mattress made of?	Cotton	1	CHMAT6A
		Sponge	2	
		Greass	3	
		Kapoak	4	
		No matress	5	
		Other (Specify)	9	
G19	What is your child's pillow made of?	Cotton	1	CHPIL6A
		Sponge	2	
		Grass	3	
		Kapoak	4	
		Cloth	5	
		No pillow	6	

		Other (Specify)	9	

Section 4. Allergy Maternal Characteristics

G20	Have you had wheezing or whistling in your chest in the last 1 year?	Yes	1	→ G35	MOWHZ6A
		No	2	→ G36	
G21	How many times in the last year have you had an attack of wheezing?	0	1		MOWHFR6A
		1-3	2		
		4-12	3		
		>12	4		
G22	Have you had asthma in the last 1 year?	Yes	1	→ G37	MOAS6A
		No	2	→ G38	
G23	Was this confirmed by a doctor?	Yes	1		MOASSDR6A
		No	2		
G24	Has the baby's father had wheezing or whistling in the chest in the last 1 year?	Yes	1		FAWHEZ6A
		No	2		
G25	Has the baby's father had asthma in the last 1 year?	Yes	1	→ G40	FAAS6A
		No	2	→ G41	
		NA	9		
G26	Was this confirmed by a doctor?	Yes	1		FAASDR6A
		No	2		
G27	In the last 1 year have you had hay fever?	Yes	1		MOHAY6A
		No	2		
G28	In the last 1 year has the baby's father had hay fever?	Yes	1		FAHAY6A
		No	2		
		NA	9		
G29	Have you had eczema in the last 1 year?	Yes	1		MOEZC6A
		No	2		
G30	Has the baby's father had eczema in the last 1 year?	Yes	1		FAEZC6A
		No	2		
		NA	9		
		No	2		

Section 5. Housing characteristics

G31	What type of roof does your house have?	Thatched	1	GROOF6A
		Corrugated iron	2	
		Other (specify)	9	
G32	What are the walls of your house made of?	Wood	1	GWALL6A
		Wood and grass	2	
		Cement	3	
		Brocket	4	
		Bricks	5	
		Corrugated iron	6	
		Other (specify)________	9	
G33	How many rooms does your house have? **(observe and fill the no of rooms)**	[ ]		GROOM6A
G34	What type of floor does your house have?	Cement	1	GFLOOR6A
		Wood	2	
		Bricks	3	
		Mud	4	
		Other (specify)_____________	9	
G35	Is the floor covered by any material?	Yes	1	GCOVER6A
		No	2	

G36	Where do you do most of your cooking? (tick one that applies)	Inside the house in the main living area	1	GCOOK6A
		Inside the house in a room other than the main living area	2	
		Outside the house in a separate building	3	
		Outside the house in the open air	4	
G37	How often do you use the following for cooking?			

Fuel	Never	Sometimes	Every day
1. Charcoal	1	2	3
2. Wood	1	2	3
3. Leaves	1	2	3

	4. Dung	1	2	3	GFUEL6A
	5. Nafta/Lanba	1	2	3	
	6. Gas	1	2	3	
	7. Electricity	1	2	3	
	9. Other	1	2	3	

G38

Which of the following animals do you or your household keep? (Multiple answers possible)

Animal	Not available	Inside	Outside
1. Cat	1	2	3
2. Dog	1	2	3
3. Hen	1	2	3
4. Cow/ox	1	2	3
5. Sheep	1	2	3
6. Horse	1	2	3
7. Pig	1	2	3
8. Goat	1	2	3
10. mule/donkey	1	2	3

GANIM6A

		9 Other	1	2	3	

G39	What is your main source of drinking water? **(Tick one which applies)**	Piped into compound	1	GWATER6A
		Piped outside compound	2	
		Open well or spring	3	
		Covered well or spring	4	
		River, pond or dam	5	
		Rainwater	6	
G40	What type of toilet facility do you use? **(Tick one which applies)**	Flush toilet	1	GTOILET6A
		Ventilated improved pit	2	
		Traditionnel pit toilet	3	
		None/bush/field	4	
G41	Is your latrine connected with the sewage system, a ditch, the river, or a well?	Sewage	0	
		Ditch	1	
		River	2	
		Well	3	
		Don’t know	4	
G42	Hand washing practice after toilet.	Always	1	
		Sometimes	2	
		Never	3	

G43	How do you wash your hands after going to the bathroom – with water only or with soap and water?	Water	1	
		Soap and water	2	
G43A	If with soap and water, do you use soap always, sometimes, or never?	Always	0	
		Sometimes	1	
		Never	2	
G44	Hand washing practice before meal.	Always	1	
		Sometimes	2	
		None	3	
G44A	How do you wash your hands before eating – with water only or with soap and water?	Water	0	
		Soap and water	1	
		Always		
G44B	If with soap and water, do you use soap always, sometimes, or never?	Sometimes		
		Never		

G45	What is your favorite fruit that you eat?	Fruit __________		
G45A	Do you wash your fruits before eating - always, sometimes, or never?	Always	0	
		Sometimes	1	
		Never	2	
G46	Do you eat raw meat	Yes	0	
		No	1	
G47	Do you drink raw milk	Yes	0	
		No	1	
G48	Do you walk barefoot - always, sometimes, or never?	Always	0	
		Never	1	
		Sometimes	2	
G49A	When you are at home do you prefer to use sandals or shoes?	Sandals	0	
		Shoes	1	
		Does not use any	2	
G49B	In which activities of the day are you barefoot?	A- Activity: ………..…………………… ……………. B- Activity: ………..…………………… …………….		

		C-Activity: ………..……………………………………….			
G50	Finger nail trimming	Trimmed		1	
		Not trimmed		2	
G51	Do you bath in the in your local river	Always		0	
		Sometimes		1	
		Never		2	
G52	How do you dispose your waste?	Pit		1	GSAND6A
		Open field		2	
		Burning		3	
		Garbage bin		4	
		Other(Specify)_______		9	
G53	Do you use any of the following insecticides in your house? **(Multiple answers possible)**	DDT	Yes	1	GINSE6AA
			No	2	
		Malathion	Yes	1	GINSE6AB
			No	2	
		Cockroch spray	Yes	1	GINSE6AC
			No	2	
		Rat poison	Yes	1	
			No	2	

		Flea and tick spray	Yes	1	
			No	2	
		Application of dung	Yes	1	GINSE6AD
			No	2	
		Other(specify)	Yes	1	GINSE6AE
			No	2	
G71	Where do you place insecticides in your house? (observe)	Out of reach of children		1	PROT6A
		Within reach of children		2	

6. Child nutritional status

**G54**	Do you eat plant food such as fruits Beans, clams, green vegetables, soybeans?	Yes	1	
		No	2	
**G55**	If yes, how often do you these plant foods per a week?	a) once<br>b) twice<br>c) more than twice		
**G56**	Do you eat meat?	Yes	1	
		No	2	
**G57**	If yes, how often do you eat per a week?	a) once<br>b) twice<br>c) more than twice		

7. Exclusion Criteria

G58	Are you currently taking any anthelminthic drugs?	yes	1	
		No	2	
G59	Have you taken iron supplements drugs within 3 months?	Yes	1	
		No	2	
G60	Have you been recently treated for infection with *H.Pylori*?	Yes	1	
		No	2	
G61	Do you have recently taken any antibiotics	Yes	1	
		No	2	
G62	Have you taken antihistamine drugs for the last fivedays(Bromphemiramine,Cetirizine,chlorphimiramine)	Yes	1	
		No	2	

Annex-XV: Questionnaire – Amharic version

አሁን ስል□□ ቀጣይ ስምናደርገው ምርመራ ይረዳን ዘንድ የሚከተሉትን □□ቱ□ች ስለምንጠ□ቀወት ባክዎን ባለፉት ቲ□ያቶች ውስጥ ያጋጠሙወትን ከዚህ ጋር ተያያዥነት ያላቸውን ችግሮች በማስታ□ስ ትክክለኛ የሆነ ምላሽ □ክንዲሰጡን በትህትና ክንጠይቀወተስን።

1.1 ህፃኑን/ኗን የተመለከተ

G01	ህፃኑ/ኗ ከተወለደ/ች ጀምሮ በየትኛውም ጊዜ ቢሆን በ□ረቱ/ትዋ ውስጥ ሲጥ ሲጥ የሚል ወይም የፉጨት ድምፅ ኖሮት ያውቃል/□ውቃለች?	አዎን	1		WHZL6A
		□ለም	0		
G02	ባለፉት ሁለት አመ□ት በየትኛውም ጊዜ ቢሆን በ□ረቱ/ትዋ ውስጥ ሲጥ ሲጥ የሚል ወይም የፉጨት ድምፅ ኖሮት ያውቃል/□ውቃለች?	አዎን	1		WHZT6A
		□ለም	0		
G03	ባለፉት 12 ወራት ዉስጥ በሕፃኑ/ኗ ደረት ውስጥ ሲጥ ሲጥ የሚል ወይም የፉጨት ድምፅ ተሰምቶ ጸዉቃል/□ውቃለች?	አዎን	1	→ G04	WHZ6A
		□ለም	0	→ G05	
G04	ባለፉት 12 ወራት ህፃኑ/ኗ ደረት ውስጥ ሲጥ ሲጥ የሚል ወይም የፉጨት ድምፅ ተሰምቶ የነበረዉ ስንት ጊዜ ነበር?	0	0		WHZFRQ6A
		1-3	1		
		4-12	2		
		ከ13 በላይ	3		
G05	ህፃኑ/ኗ ከተወለደ/ች ጀምሮ በየትኛውም ጊዜ ቢሆን አስም ኖሮት ያውቃል/□ውቃለች?	አዎን	1		ASTL6A
		□ለም	2		
G06	ባለፉት ሁለት አመ□ት በየትኛውም ጊዜ ቢሆን ህፃኑ/ኗ አስም ኖሮት/ሯት ያውቃል/□ውቃለች?	አዎን	1		ASTT6A
		□ለም	0		
G07	ባለፉት 12 ወራት ዉስጥ ህፃኑ/ኗ አስም ኖሮት ጸዉቃል/□ውቃለች?	አዎን	1	→G08	AST6A
		□ለም	0		
**G08**	ህፃኑ/ኗ አስም እንዳለበት/ባት በሐኪም ተረፋፅ□ል?	አዎን	1		ASTHDR6A
		□ለም	0		
**G09**	ህፃኑ/ኗ ከተወለደ/ች ጀምሮ በየትኛውም ጊዜ ቢሆን በአጥንት መታጠፊያ ቦታዎቹ(ቿ) (በክርን መታጠፍያ፤ከጉልበቱ ኋላ ባለዉ መታጠፍያ፤በቁርጭምጭሚት ፊት ለፊት፤ በአንገት □ሪጸ፤ እና በአይን አካባቢ) የሚያሳክክ ሽፍ□ ወጥቶበት (ወጥቶባት) ነበር?	አዎን	1		RASHL6A
		□ለም	0		
G10	ባለፉት ሁለት አመ□ት በየትኛውም ጊዜ ቢሆን ህፃኑ/ኗ በአጥንት መታጠፊያ ቦታዎቹ(ቿ) (በክርን መታጠፍያ፤ከጉልበቱ ኋላ ባለዉ መታጠፍያ፤በቁርጭምጭሚት ፊት ለፊት፤ በአንገት □ሪጸ፤ እና በአይን አካባቢ) የሚያሳክክ ሽፍታ ወጥቶበት (ወጥቶባት) ነበር?	አዎን	1		RASHT6A
		□ለም	0		
G11	ባለፉት 12 ወራት ዉስጥ ልጁ(ልጅቷ) በአጥንት መ□ቷኝጸ ቦታዎቹ(□) (በክርን መታጠፍያ፤ከጉልበቱ ኋላ ባለ□ መታጠፍያ፤በቁርጭምጭሚት ፊት ለፊት፤ በአንገት ዙሪያ፤ እና በአይን አካባቢ) የሚያሳክክ ሽፍታ ወጥቶበት (ወጥቶባት) ነበር?	አዎን	1	→G11A	RASH36
		□ለም	0		

G11A	መልሱ አዎን ከሆነ: ሽአታዉ የነበረዉ በየትኛዎቹ ቦታዎች ላይ ነዉ?<br><br>(መልሱ ይነበብ፤ ከአንድ በላይ መልስ መስጠት ይቻላል)	በክርን መታጣኛጸ	1<br>0		RASH6AA
		ከጉልበትዎ ኋላ	1<br>0		RASH6AB
		በቁር□ ም□ ሚ ት ፊትለፊት	1<br>0		RASH6AC
		ከመቀመጫ በታች	1<br>0		RASH6AD
		በአንገትዎ ዙሪያ	1<br>0		RASH6AE
		በአይንና በጆሮዎች □ሪጸ	1<br>0		RASH6AF
G12	ህፃኑ/ኗ ከተወለደ/ች ጀምሮ በየትኛውም ጊዜ ቢሆን ንፍጥ የሚያበዛ ጉንፋን፤የማያቋርጥ ማስነጠስ፤አፍንጫ ወይም ዐይንን የሚያቃጥል ሕመም ነበረበት(ባት)? (□ነኚህ ምልክቶች የ□ዩት ህፃኑ በጉንፋን ሳይያዝ መሆን አለበት)።	አዎን<br>□ለም	1<br>0		HAYFL6A
G13	ባለፉት ሁለት አመ□ት በየትኛውም ጊዜ ቢሆን ህፃኑ/ኗ ንፍጥ የሚያበዛ ጉንፋን፤የማያቋርጥ ማስነጠስ፤አፍንጫ ወይም ዐይንን የሚያቃጥል ሕመም ነበረበት(ባት)?<br><br>(□ነኚህ ምልክቶች የ□ዩት ህፃኑ በጉንፋን ሳይያዝ መሆን አለበት)።	አዎን<br>□ለም	1<br>0		HAYFT6A
G14	ባለፉት 12 ወራት ዉስጥ ልጅዎ ንፍጥ የሚያበዛ ጉንፋን፤የማያቋርጥ ማስነጠስ፤አፍንጫ ወይም ዐይንን የሚያቃጥል ሕመም ነበረበት(ባት)? (□ነኚህ ምልክቶች □□ዩት ህፃኑ በጉንፋን ሳይያዝ መሆን አለበት)።	አዎን<br>□ለም	1<br>0		HAYF6A
አሁን ህፃኑን/ኗ በ□መሙ ቂ□ □ራሴ□ሞልና □መሳሰሉትን □ህመም ማስ□ቶሻ እንደወሰዐ □ኗጁቅ- □ለሁ።					
G15	□ራሴ□ሞል ከአስፕሪን ጋር አንድ ነው?	አዎን<br>□ለም	1<br>0	 →G17	PADIF6A
G16	ከነዚህ ከሁለቱ መካከል ፓራሴታሞልና አስፕሪንን ልትለይልን ትችያለሽ ?	ትክክለኛ መለጸ<br>□ተሳሳተ መለጸ	1<br>0		PADFA6A
G17	ሕጻኑ(ኗ) ባለፈዉ ዓመት ፓራሴታሞል/ፓናዶል ወስ□(□) ጸ□ ቃል(ታዉቃለች)?	አዎን<br>□ለም	1<br>0	→G18<br>→G20	PARA6A
G18	ህፃኑ/ኗ በላፈው ወር ስንት የፓራሴ□ሞል ወጁም<br><br>ፓናዶል ኪኒኖች ወሰዶዋል? (በቁዓር ጁቶለን)	[ ] [ ] ወስዶዋል/ለች።			PARAFR6A
G19	□ራሴ□ሞል ለሕጻኑ(ኗ) የሰጡበት □ሕመም ምልክቶች/በሽ□ዎች ከሚከተሉት የትኞቹ ናቸው?<br><br>(መልሱ ይነበብ፤ ከአንድ በላይ መልስ መስጠት ይቻላል)	ራስ ምታት	አዎን<br>□ለም	1<br>0	PAHED6A
		ትኩሳት	አዎን<br>□ለም	1<br>0	PAFEV6A
		ወበ	አዎን<br>□ለም	1<br>0	PAMAL6A
		ጉንፋን	አዎን<br>□ለም	1<br>0	PACOLD6A
		ብርት	አዎን<br>□ለም	1<br>0	PABIRD6A

		ሳል	አዎን	1		PACOU6A
			□ለም	0		
		ሲዓ ሲዓ ሲልበት/ባት	አዎን	1		PAWHEZ6A
			□ለም	0		
		የትንፋሽ ማጠር	አዎን	1		PASOB6A
			□ለም	0		
		ማስነጠስ/ እንደ ንፍጥ ጸለ በአፍንጫ ሲወርት/የአይን ማሳከክ ሲኖር	አዎን	1		PASNEZ6A
			□ለም	0		
		□ቆ□ ሽአ□ በመ□ቷኝጸ አካባቢዎች ሲኖር	አዎን	1		PARASH6A
			□ለም	0		
		ሌላ (ጁቆለን) ______________________				PAOTHE6A
G20	በሚኖሩበት አካባቢ □ራሴታሞልን በቅርበት ያገኙ□ል?	አዎን		1		PARAV6A
		□ለም		0		
G21	□ርስ- □ራሴታሞልን ለመግዛት ዋጋውን ይችሉታል?	አዎን		1		PAFORD6A
		□ለም		0		
G22	አስፐሪን ለልጅዎ ሳለመስጠት ይሞክራሉ?	አዎን		1		ASAVOD6A
		□ለም		0		
G23	አስፐሪን መውሰድ የሌለባቸው ሰዎች አሉ?<br><br>(መልሱ ይነበብ፤ ከአንድ በላይ መልስ መስጠት ይቻላል)	ሕፃናት	አዎን	1		ASCHIL6A
			□ለም	0		
		ጨጓራ ያለባቸው	አዎን	1		ASGAS6A
			□ለም	0		
		አስም ያለባቸው	አዎን	1		ASASTH6A
			□ለም	0		
		የአፍንጫ አስም ያለባቸው	አዎን	1		ASHAY6A
			□ለም	0		
		አላውቅም	አዎን	1		ASAVDK6A
			□ለም	0		
		ሌላ (ጁቆለን)__________________				
G24	□ርስዎ ለልጅዎ ለመስጠት የሚመርጡት አስፐሪንን ነው ወይንስ □ራሴታሞልን?	አስፐሪን		1		ASPREF6A
		□ራሴ□ሞል		2		
		እንደ ሁኔ□ ዉ፡		3		
		ምንም ምርጫ የለኝም		4		
G25	ህፃኑ/ኗ ለማንኛውም አይነት<br><br>መትኃኒት ለየትኛውም አይነት በሽ□<br><br>በሕክምና በቅርብ ጊዜ ታዞለት/ላት<br><br>ያውቃል(ለች)?<br><br>**(ይህን ጥያቄ ለማኝኛውም አይነት በሽ□ □ታዘዘለትን/ላትን መድሃኒት ያካትታል ነገር ግን ፓራሴንታሞል/ፓናዶልን ወይም አስፐሪንን አይጨምርም፡፡)**	አዎን		1	→G25A	ANTIB6A
		□ለም		0	→G26	

G25A	መልስዎ አዎ ከሆነ የሚወስዱትን መድሃኒት አይነቱንና ስሙን በማየት ይሞላ::	1.------------------------------			BANTA6A
		2.------------------------------			BANTB6A
		3.------------------------------			BANTC6A
G26	ህፃኑ/ኗ ባለፉት ስድስት ወራት ለሆድ ትላትል መከላከያ መድሃኒት ወስዶአል?<br><br>(የሆድ ትላትል መከላከያ ሲባል በዋነኛነት በጤና ባለሙጳ በአመት ሁለት ጊዜ ቤት ለቤት በነፃ የሚ□□ል ማለት ነው::)	አዎን	1		DEWOR6A
		□ለም	0		
G27	በቤት ውስጥ ምንያህል ሰዎች ይኖራሉ?	[ ] [ ]			PEOP6A
G28	ህፃኑን/ኗ ስንት □ላቅ ወንድምና □ህቶች በሕይወት አሉት/አሏት?	[ ] [ ]			SIBS6A
G29	ህፃኑ/ኗ በሚኖርበት/በምትኖርበት ቤት ውስጥ ሲጋራ/ትምባሆ የሚያጨስ ሰው አለ?	አዎን	1	→G29A	HCIGR6A
		□ለም	0	→G30	
G29A	መልሱ አዎ ከሆነ የሚያጨስ ሰው<br><br>ብዛት ጠይቀሽ መዝግቢ	[ ] [ ]			HCIGRN6A
G30	ህፃኑ/ኗ በምን ላይ ነዉ የሚተኛ<br><br>ዉ(የምትተኛ□)?	አልጋ	1		CHSLP6A
		መ□ብ	2		
		ወለል	3		
		ፀባ	4		
		ፗኘነ	5		
		ሌላ (ጁፅለ□)	9		
G31	ህፃኑ/ኗን ለመኝ□<br><br>የሚጠቀሙዉ/የምትጠቀመዉ አልጋ<br><br>ከሆነ የተሠራው ከምንድ ነው?	ከሸቦ	1		CHBED6A
		ከ□ንጨት	2		
		ከቦንዳ	3		
		ከገመድ	4		
		ከቁርበት	5		
		አልጋ የለኝም	6		
		ሌላ (ጁፅለ□)	9		

G32	ህፃኑ/ኗን ለመኝ□ የሚጠቀሙዉ/የምትጠቀመዉ ፍራሽ ከሆነ የተሠራው ከምንድ ነው?	ከጥጥ	1	CHMAT6A
		ከስፖንጅ	2	
		ከሳር	3	
		ከአበባ የሚገኝ ጥጥ መሰል ነገር	4	
		ፍራሽ የለኝም	5	
		ሌላ (ጁፅለ□	9	
G33	ህፃኑ/ኗን ለመኝ□ የሚጠቀሙዉ/የምትጠቀመዉ ትራስ ከሆነ የተሠራው ከምንድ ነው?	ከጥጥ	1	CHPIL6A
		ከስፖንጅ	2	
		ከሳር	3	
		ከአበባ የሚገኝ ጥጥ መሰል ነገር	4	
		ፊ ርቅ ወጁም ልብስ	5	
		ከጨርቅ የተሰራ ትራስ	6	
		ትራስ የለውም/ላትም	7	
		ሌላ (ጁፅለ□	9	

1.2 የህፃኑ/ኗ □ናትን/አባትን □ተመለከተ

**G34**	ባለፉት 12 ወራት በደራትዎ ዉስጥ ሲጥ ሲጥ የሚል ወይም የፉጨት ድምፅ ነበረብዎት?	አዎ	1	→G35	MWHZ6A
		□ለም	0	→G36	
**G35**	ባለፉት 12 ወራት በደራትዎ ዉስጥ ሲጥ ሲጥ □ሚል ወይም የፉጨት ድምፅ ተሰምቶዎት የነበረዉ ስንት ጊዜ ነበር?	0	0		MWHFR6A
		1-3	1		
		4-12	2		
		ከ13 በላይ	3		
**G36**	ባለፉት 12 ወራት አስም ነበረብዎት?	አዎ	1	→G37	MOAS6A
		□ለም	0	→G38	
**G37**	□ርስዎ አስም □ንዳለብዎት በሐኪም ተረፉ□ል?	አዎ	1		MASDR6A
		□ለም	0		
**G38**	ባለፉት 12 ወራት □ልፃ (ልፀ ቷ) አባት በ□ረ□ቸው □ስዓ ሲዓ ሲዓ □ሚል ወጁም የፉጨት ድምፅ ነበረባቸው?	አዎ	1		FWHZ6A
		□ለም	0		
		አይመለከትም	9		
G39	ባለፉት *12* ወራት የልጁ(ልጅቷ) አባት አስም ነበረባቸዉ?	አዎ	1	→G40	FAAS6A
		□ለም	0	→G41	
		አይመለከትም	9		
G40	□ልፃ (ልፀ ቷ) አባት አስም □ንዳለባቸው በሐኪም	አዎ	1		FASDR6A

	ተረፋ□ል?	□ለም	0	
G41	ባለፉት 12 ወራት ዉስጥ ንፍጥ የበዛበት ጉንፋን፤ የማያቋርጥ ማስነጠስ፤ አፍንጫ ወይም ዓይን ማቃጠል ነበረብዎት?	አዎ	1	MOHAY6A
		□ለም	0	
G42	ባለፉት 12 ወራት ዉስጥ የልጁ(ልጅቷ) አባት፤ ንፍጥ የበዛበት ጉንፋን፤ የማያቋርጥ ማስነጠስ፤ አፍንጫ ወይም ዓይን ማቃጠል ነበረባቸዉ?	አዎ	1	FAHAY6A
		□ለም	0	
		አይመለከትም	9	
G43	ባለፉት 12 ወራት የሚያሳክክና ፤በተለጁም የአጥንት መ□ቷኘጸ አካባቢዎች ጸሉትን የሰውነት ክፍሎችን/ለምሳሌ የክንድ፤ ከጉልበት በስተኋላ □□ኘ ቆዳዎችን/ □ሚጸቷቃ □ቆ□ ሽአ□ ነበረብዎት?	አዎ	1	MOEZCA6A
		□ለም	0	
G44	ባለፉት 12 ወራት የልጁ(ልጅቷ) አባት ፤በተለይም የአጥንት መ□ቷኘጸ አካባቢዎች ጸሉትን □ሰዉነት □ኢሎችን/ለምሳሌ የክንድ፤ ከጉልበት በሰተኋላ □□ኘ ቆዳዎችን/ □ሚጸቷቃ □ቆ□ ሽአ□ ነበረባቸዉ?	አዎ	1	FAEZC6A
		□ለም	0	
		አይመለከትም	9	
G45	በላፈው ዓመት ፓራሴ□ሞል/ፓናዶል ወስ□ዉ ጸዉቃሉ?	አዎ	1	MOPAR6A
		□ለም	0	
G46	በላፈው ወር ሰንት የፓራሴ□ሞል ወጁም ፓናዶል ኪኒኖች □ቷ□ል?	[ ] [ ] ወስዳለች::		MOPAFR6A
G47	ማንኛውም አይነት መድኃኒት ለየትኛዉም አይነት በሽታ በሕክምና በቅርብ ጊዜ ታዞልዎት ጸዉቃል? **(ይህን ጥያቄ ለማኛኛውም አይነት በሽታ የታዘዘልዎትን መድሃኒት ያካትታል ነገር ግን ፓራሴንታሞል/ፓናዶልን ወይም አስፐሪንን አይጨምርም::)**	አዎን	1	MANTIB6A
		□ለም	0	
G48	መልስዎ አዎ ከሆነ የሚወስዱትን መድሃኒት አይነቱንና ስሙን በማየት ይሞላ::	1.-----------------------------		MANTA6A
		2.-----------------------------		MANTB6A
		3.----------------------------		MANTC6A

1.3 ቤትዎን የተመለከተ

G49	የቤትዎ ጣራ የተሰራው ከምንድን ነው?	ሣር	1	GROOF6A
		ቆርቆሮ	2	
		ሌላ (ጁቶስ□)	9	
G50	ግድግዳው ከምን የተሠራ ነው?	እንጨትና ጭቃ	1	GWALL6A
		እንጨት፣ ጭራሮና ሳር	2	
		ድንጋይና ሲሚንቶ	3	
		ብሎኬት	4	
		ጡብ	5	
		ቆርቆሮ	6	
		ሌላ (ጁቶስ□)	9	
G51	የህፃኑ/ኗ መኖሪያ ቤት ወለል የተሰራው ከምንድን ነው?			GFLOOR6A
		ከሲሚንቶ	1	
		ከጣውላ ወይም □ንጨት	2	
		ከሸክላ	3	
		ከአፈር	4	
		ሌላ(ጁፅለ□)	9	
G52	የህፃኑ/ኗ መኖሪያ ቤት ወለል በምንጣፍ ወይም በሌላ ነገር ተሸፍኗል?	አዎን	1	GCOVER6A
		□ለም	0	
G53	ቤተሰቡ ምግብ በብዛት የሚያበስሉት የት ነው? **(አንዱ ላይ ብቻ ምልክት አድርጊ)**	በዋናው ቤት ውስጥ	1	GCOOK6A
		□ቤት ውስጥ ሆኖ ከዋናው ቤት ሌላ	2	
		ከቤት ውጭ ማዕድ ቤት	3	
		ከቤት ውጭ ክፍት ቦ□	4	
G54	የህፃኑ/ኗን ቤተስበ ከሚከተሉት የማገዶ ዓይነቶች ምግብ ለማብሰል በየስንት ቴ□ው □ንድሚቷቀሙ ቢቶቂናል?			GFUEL6AA GFUEL6AB

ማገዶ/ነዳጅ	አልፎቀምም	አንዳንድ ቴ□	ምየቀኑ
1. ከሠል	1	2	3
2. □ንጨት	1	2	3

3. ቅቷል	1	2	3	GFUEL6AC
4. ኩበት	1	2	3	GFUEL6AD
5. ናፍጣ/ላንባ	1	2	3	GFUEL6AE
6. ቡ□ፋ□	1	2	3	GFUEL6AF
7. መብራት/ኮርነቲ	1	2	3	GFUEL6AG
9. ሌላ (ጁፅለ□)	1	2	3	GFUEL6AH

G55 የህፃኑ/ኗን ቤተሰበ ከሚከተሉት የማገዶ ዓይነቶች ምግብ ከማብሰል ውጭ ለሌላ ጉዳይ ይጠቀማል (ለምሳሌ ለሙቀትና ለመብራት)?

ማገዶ/ነዳጅ	አልጠቀምም	አንዳንድ ቲ□	በየቀኑ	
1. ከሠል	1	2	3	GFUEL6AAA
2. □ንጨት	1	2	3	GFUEL6AAB
3. ቅቷል	1	2	3	GFUEL6AAC
4. ኩበት	1	2	3	GFUEL6AAD
5. ናፍጣ/ላንባ	1	2	3	GFUEL6AAE
6. ቡ□ፋ□	1	2	3	GFUEL6AAF
7. ባትሪ ድንጋይ	1	2	3	GFUEL6AAG
8. መብራት/ኮርነቲ	1	2	3	GFUEL6AAH
9. ሌላ (ጁፅለ□)	1	2	3	GFUEL6AAI

G56 የህፃኑ/ኗን ብተሰብ ከሚከተሉት □ንሳሳትና ከብቶች ዓይነቶች የትኞቹ አሏችው?

□ንስሳ	በቤት ውስጥ ጁቷበቃሉ/ጸት ራሉ	ከቤት ውጪ ጁቷበቃሉ
1. ድመት	1	2

		**2. ዉሻ**	**1**	**2**	GANIM6AA
		**3. □ሮ**	**1**	**2**	GANIM6AB
		**4. ላም/በሬ**	**1**	**2**	GANIM6AC
		**5. በፅ**	**1**	**2**	GANIM6AD
		**6. □ረስ**	**1**	**2**	GANIM6AE
		**7. አ□ል**	**1**	**2**	GANIM6AF
		**8. በቅሎ/አህያ**	**1**	**2**	GANIM6AG
		**9. ሌላ**	**1**	**2**	GANIM6AH
					GANIM6AI
G57	**የህፃኑ/ኗን ቤተስብ የመጠጥ ውሃ በዋንኝነት የሚያገኙት ከየት ነው?**	**በግቢው ውስጥ ከሚገኝ ቧንቧ**	**1**		WAT6A
		**ከግቢ ውጪ ከሚገኝ ቧንቧ**	**2**		
		**ከጉድጓድ ወይም ምንጭ**	**3**		
		**ከተጠበቀ ጉድጓድ ወይም ምንጭ**	**4**		
		**ከወንዝ፣ ከኩሬ፣ከጉድጓድ**	**5**		
		**ከዝናብ ውሃ**	**6**		
G58	**የህፃኑ/ኗን ቤተስቡ የሚገለገልበት መፀዳጀ ቤት ምን አይነት ነው?**	**በውሃ የሚሰራ ሽንት ቤት**	**1**		SANIT6A
		**ሽ□ አልባ መ□□ጽ**	**2**		
		**የተለመደ ዓይነት የሽንት ቤት ቶትጓት**	**3**		
		**ሜ□ ላጁ ወጆም □ ካ**	**4**		
		**በየቀኑ**	**2**		
		**ቢያንስ በሳምንት አንድ ጊዜ**	**3**		
		**ቢያንስ በ15 ቀን አንድ ጊዜ**	**4**		
		**በበዓል ቀን ወይንም ለየት ባለ ቀን**	**5**		
		**አላውቅም**	**6**		
		**መልስ መስጠት አልፈለኩም**	**7**		
G59	**የህፃኑ/ኗ ቤተሰብ ቆሻሻ ለመጣያነት የሚገለገሉበት ምንድን ነው?**	**በቶትጓት ዉስዓ**	**1**		GSAND6A
		**ሜ□ ላጁ**	**2**		

		አንድ ላይ ሰብስቦ በማቃጠል	3	
		በቆሻሻ መ□ጸ □ቃ	4	
		ሌላ (ጁቶስን)	9	
G60	የህፃኑ/ኗ ቤተሰብ ከሚከተሉት የተባይ ማዓ ኘጸ መት□ኒቶች በቤቱ<br>ውስጥ የትኛውን ይጠቀማሉ?<br>**(ከአንድ በላይ መልሰ መስጠት**<br>**ይቻላል) (መልሱ ይነበብ)**	ዲዲት	1<br>0	GINSE6AA
		ማላ□ይን	1<br>0	GINSE6AB
		ፍሊት	1<br>0	GINSE6AC
		ሌላ (ጁፅለ□)	1<br>0	GINSE6AD
G61	□ንደ ፊሊት የመሳሰሉት ጸረተባይ መድሀኒቶች የት ነው የሚያስቀምጡት?**(ስፍራዉን □ንዲያሳዩሽ ጠይቂያቸዉ)**<br><br>**አሁን ቤት ዉስዓ □ረተባጁ መት□ኒቶች ከሌላቸው ቢኖራቸው ኖሮ የት □ንደሚያስቀምጡ ጠይቂያቸው**	ልጆች ሊደርሱበት<br>በሚችሉበት ቦ□	1	PROT6A
		ልጆች ሊደርሱበት<br>በማይችሉበት ቦ□	0	
